# Can prosocial values improve brain health?

**DOI:** 10.3389/fneur.2023.1202173

**Published:** 2023-06-05

**Authors:** Agustin Ibanez, Diana Matallana, Bruce Miller

**Affiliations:** ^1^Latin American Institute for Brain Health (BrainLat), Universidad Adolfo Ibanez, Adolfo Ibanez University, Santiago, Chile; ^2^Cognitive Neuroscience Center (CNC), Universidad de San Andrés, and National Scientific and Technical Research Council (CONICET), Buenos Aires, Argentina; ^3^Global Brain Health Institute, University of California, San Francisco, San Francisco, CA, United States; ^4^Trinity College Dublin, Dublin, Ireland; ^5^Pontificia Universidad Javeriana, Instituto de Envejecimiento, Bogotá, Colombia; ^6^Memory and Cognition Center, Intellectus, Hospital Universitario San Ignacio, Bogotá, Colombia; ^7^Memory and Aging Center, University of California, San Francisco, San Francisco, CA, United States

**Keywords:** prosocial values, prosociality, social cognition, brain health, Allostasis, empathy, cooperation, moral cognition

## Abstract

Prosocial values play a critical role in promoting care and concern for the well-being of others and prioritizing the common good of society. Evidence from population-based reports, cognitive neuroscience, and clinical studies suggests that these values depend on social cognition processes, such as empathy, deontological moral cognition, moral emotions, and social cooperation. Additionally, indirect evidence suggests that various forms of prosocial behaviors are associated with positive health outcomes at the behavioral, cardiovascular, immune, stress-related, and inflammatory pathways. However, it is unclear whether prosociality can positively influence brain health outcomes. In this perspective, we propose that prosocial values are not only influenced by brain conditions but could also potentially play a role in protecting brain health. We review studies from various fields that support this claim, including recent reports of prosociality-based interventions impacting brain health. We then explore potential multilevel mechanisms, based on the reduction of allostatic overload at behavioral, cardiovascular, immune, stress-related, and inflammatory levels. Finally, we propose potential prosociality-based interventions for improving brain health in at-risk populations, such as psychiatric and neurological patients, and individuals exposed to poverty or violence. Our perspective suggests that prosocial values may play a role in promoting and maintaining healthy brains.

## Prosocial values and social cognition

Prosocial values are defined as the beliefs, attitudes, and behaviors that promote the well-being and welfare of others, with an emphasis on cooperation, helping, sharing, and altruism (1). Prosocial values promote care and concern for the welfare of others and are critical for prioritizing the common good of society at large. These include cognitive, moral, and socioemotional processes that prioritize the well-being of others and society over one’s own interests. These values are important for building strong and healthy communities, promoting positive social interactions, and creating a sense of social connectedness.

Studies in economics and psychology suggest that prosocial behavior improves common goods, such as increased cooperation and trust among individuals, better outcomes for collective action problems, and improved resource management. This effect has been particularly observed in challenging contexts like the pandemic (2–6). In social decision-making contexts, individuals tend to contribute more to a common pool of resources when they believe that others are also making contributions (7). Similarly, from childhood to adulthood, prosocial behavior can increase levels of cooperation and trust, leading to improved relationships and increased cooperation in both social and economic contexts (8–10). Overall, prosocial behavior can have a positive impact on common goods and improve the overall well-being of society.

Population-based reports, cognitive neuroscience, and clinical studies have linked prosocial behaviors with different social cognition processes, such as empathy, deontological moral cognition, moral emotions, and social cooperation. Empathy, or the ability to understand and share the feelings of others (11), has been closely linked to prosocial behavior (12). People who score higher on empathy measures are more likely to engage in behaviors that benefit others. Similarly, compassion involves an emotional response of understanding, caring, and alleviating the suffering of others. While empathy focuses on sharing and mirroring emotions, compassion goes further by involving active help and support. Deontological moral cognition refers to the beliefs and values supporting the groups’ benefits, even at the expense of potential negative individual consequences. It also emphasizes adherence to rules, duties, or principles, regardless of the consequences or outcomes. These moral processes have been found to play a significant role in shaping prosocial behavior (13). Moral emotions, such as guilt, shame, or counter-empathic emotions (envy, Schadenfreude), are complex affective processes linked to the ethical aspects of one’s actions or thoughts and have also been linked to prosocial behavior (14). Although typically seen as negative, counter-empathic emotions can promote prosocial behavior (15). Envy can inspire self-improvement or goal-setting, leading to positive outcomes for individuals and society. Meanwhile, Schadenfreude can foster social cohesion by highlighting unfairness or promoting group norms as a form of social regulation. Finally, social cooperation, or working together with others toward a common goal (i.e., sharing resources, coordinating efforts, establishing trust within a group) has been shown to be a key factor in prosocial behavior (16). Nevertheless, despite the evidence from real-life settings and neurocognitive correlates, a remaining question is whether prosociality can positively impact brain health outcomes, which refers to measures of cognitive, emotional, motor and neurological well-being, that can be influenced by various factors, including genetics, environment, lifestyle, or access to healthcare.

## Could prosocial values play an unrecognized role in brain health?

According to the World Health Organization, brain health refers to the state of brain functioning across cognitive, sensory, social–emotional, behavioral and motor domains, allowing a person to realize their full potential over the life course, irrespective of the presence or absence of disorders (17). Various determinants affect brain development, adaptation, and response to stress and adversity, including physical and mental health, safe environments, security, lifelong learning, social connections, and access to quality services.

Multiple brain diseases (psychiatric and neurological conditions) compromise the core cognitive components of prosociality (18, 19), including empathy (11, 18, 20, 21), moral cognition (18, 22–24), moral emotions (15, 25), and social cooperation (18, 26–29). Conversely, it is not well understood if prosocial habits can induce brain changes. Can prosocial values not only be influenced by brain conditions but also play a crucial, yet overlooked, role in maintaining brain health?

Prosocial behaviors and social cognition have been associated with health in a variety of ways. Engaging in prosocial behaviors has been shown to have a positive impact on mental health (30). Prosocial activities are more likely to experience feelings of happiness, well-being, and social connectedness, which can help to reduce stress and anxiety (31). Even engaging in prosocial behaviors improve mood and reduce symptoms of depression and anxiety. For instance, prosocial behavior mitigates the adverse effects of stressors on emotional well-being, suggesting that it could be an effective stress-coping strategy (32). A statewide population-based study demonstrated that people who engage in volunteer work experience positive impacts on physical health, life satisfaction, social well-being, and reduced depression (33).

At physiological levels, prosociality has been linked with multimodal health. At cardiovascular level and physiological stress responses, prosocial behavior has been linked to improved health (34). For example, prosocial activities are associated with lower levels of inflammation (proinflammatory cytokine activity) (35), which is a risk factor for heart disease and other chronic diseases; and to lower blood pressure (36), which is another important factor for cardiovascular health. The immune system can also be negatively impacted by lack of prosocial behavior (37). Studies have found that individuals who engage in prosocial activities have stronger immune system, which can help to protect against a variety of health conditions (38). Neurohormonal circuitry in caregivers, particularly oxytocin and progesterone, may contribute to the health and longevity benefits associated with helping others due to their stress-buffering and restorative properties (38). Engaging in prosocial activities has been shown to reduce levels of cortisol (39) and inflammation (40), which are key factors in many chronic health conditions. Inflammation protects the overall health as part of the immune response to infection, injury, or harmful substances. While acute inflammation is crucial for protecting the body and maintaining health, chronic inflammation can have detrimental effects on health. Inflammation can cause damage to the body’s tissues and contribute to the development of conditions such as heart disease, arthritis, and cancer. By reducing inflammation, prosocial behaviors help to reduce the risk of multiple conditions and improve overall health.

Prosociality-based interventions are designed to enhance empathy, compassion, cooperation, and other prosocial traits that contribute to the well-being of others and foster positive social interactions. Such intervention can take various forms, such as educational programs, group activities, mindfulness practices, or cognitive-behavioral therapies. Some of these prosociality-based interventions have begun to show improvements in brain health (41–43). A longitudinal study (41) found that training in socio-affective and socio-cognitive skills resulted in specific changes in brain morphology among healthy adults, correlating with improvements in cognition and prosociality, and structural plasticity in social brain networks. Socio-affective training reduces experienced negative affect when processing images depicting human suffering and increases activation in the right supramarginal gyrus when confronted with negative stimuli (42). Another study discovered that induced prosocial skills, such as compassion for others and the ability to take another’s perspective, are associated with short-term changes in leukocyte telomere length (LTL) and accompanying changes in plasticity of social brain areas (43). Eudaimonic and hedonic lifestyles represent distinct approaches to health and well-being, with eudaimonic well-being focusing on pursuing meaning, personal growth, and self-realization, while hedonic well-being emphasizes seeking pleasure and avoiding pain. Eudaimonic well-being can improve mental health, enhance immune function, and increase longevity, while hedonic well-being can reduce stress, improve cardiovascular health, and enhance social connections (44). In brief, these studies suggest that behavioral changes related to prosociality induce short-term changes associated with improved brain health.

**Figure 1:**
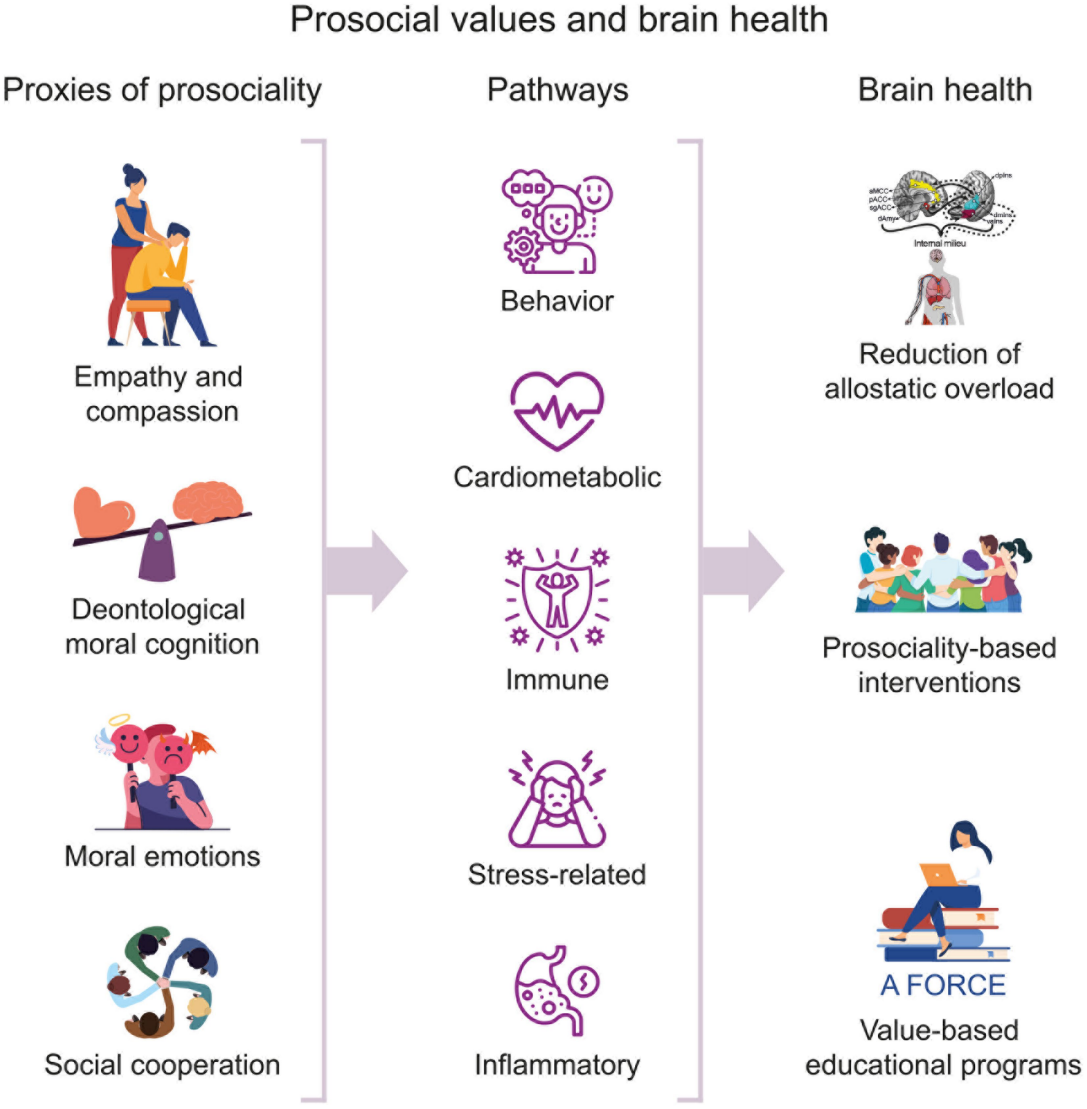
The relationship between proxies of prosocial values and brain health through biological pathways. This figure illustrates the proposed hypothesis that proxies of prosocial values, which include social cognitive and affective processes (empathy, deontological moral cognition, moral emotions, and social cooperation), can impact various behavioral and biological pathways. These encompass cardiometabolic, immune, stress-related, and inflammatory processes, which can subsequently enhance brain health by reducing allostatic overload. Integrating prosocial values into educational models (e.g., the A FORCE model), interventions, and public health policies may contribute to improved brain health, particularly in vulnerable populations.

In summary, the available although still emerging indirect evidence suggests that prosocial behavior and social cognition can positively impact health at multiple levels, including behavioral, cardiovascular, immune, stress-related, inflammatory pathways, and brain health (Figure 1). These different effects could be understood as a mechanism for reducing the environmental demands and stress. Allostatic load refers to the cumulative wear and tear on the body’s stress systems that occurs over time as a result of chronic exposure to stress and involves a continuous process of energy balance instantiated by the brain to anticipate, regulate, and respond to environmental demands (45, 46). Allostatic overload can lead to alterations in the brain’s neural circuitry, neurotransmitter systems, and inflammatory responses, which in turn can impair cognitive functions, emotional regulation, and overall mental well-being. Prosocial behavior has been linked to reduced allostatic overload (39), which can help protect the brain’s health against stress and aging and improve overall health.

## Can prosociality help to improve brain health in populations at risk?

Diverse groups, including individuals affected by negative social or environmental exposures (47), as well as psychiatric (e.g., schizophrenia, anxiety, depression, PTSD) (48), and neurological conditions (e.g., Alzheimer’s disease, frontotemporal dementia) (45, 49), constitute populations at risk impacted by allostatic overload. Psychiatric and neurological patients, and individuals exposed to stressful conditions (i.e., poverty or violence) are exposed to allostatic overload (45, 46, 50–53). Prosociality has been linked to allostatic mechanisms (39, 54–56) and prosociality-based interventions have the potential to significantly improve brain health in different populations (41–43). Promoting prosocial values and behaviors can help to reduce allostatic load, improve mood and reduce symptoms of anxiety and depression, improve cardiovascular health, and strengthen the immune system (39, 46) and even directly influence brain health.

Given these potent brain health benefits, in addition to the well know benefic promoting care and concern for the well-being of others, prosociality could be promoted in science, politics, and governmental initiatives. The Global Brain Health Institute (GBHI) has developed “A FORCE” model, a prosociality value-based learning program, which is centered around the six core values of Authenticity, Fairness, Openness, Respect, Courage, and Empathy. A FORCE involves education and training on these values and how they can be applied in daily life and in the context of brain health. Through workshops, discussions, role-playing exercises, and other interactive activities the program help students to internalize these values and understand their importance in promoting brain health. This approach could be the first step for incorporate the value of prosociality into public health policies and programs. Value-based education across the lifespan could be particularly useful in children and adolescents, as well as to understanding how prosociality can improve the long-term cumulative burden across the lifespan.

## Conclusion

In this perspective, we linked prosociality with a set of social cognitive processes and proposed that not only is it impacted by various disorders, but it also has the potential to play a role in promoting brain health. Emerging evidence supports this claim, in terms of reducing allostatic overload at behavioral, cardiovascular, immune, stress-related, and inflammatory levels. By incorporating prosocial values into education and public health policies, and developing targeted interventions to support prosocial behavior, we can help to improve brain health in populations that are particularly vulnerable to stress and adverse health outcomes.

## Data availability statement

The original contributions presented in the study are included in the article/supplementary material, further inquiries can be directed to the corresponding author.

## Author contributions

AI conceived this work and prepared the initial draft. DM and BM carefully revised the draft. All authors contributed to the contents of this article and approved the final version.

## Funding

AI is partially supported by grants ANID/FONDECYT Regular (1210195, 1210176, and 1220995); ANID/FONDAP/15150012; ANID/PIA/ANILLOS ACT210096; ANID/FONDEF ID20I10152 and ID22I10029; ANID/FONDAP 15150012; Takeda CW2680521 and the MULTI-PARTNER CONSORTIUM TO EXPAND DEMENTIA RESEARCH IN LATIN AMERICA [ReDLat, supported by Fogarty International Center (FIC) and National Institutes of Health, National Institutes of Aging (R01 AG057234), Alzheimer’s Association (SG-20-725707), Rainwater Charitable foundation – Tau Consortium, and Global Brain Health Institute].

## Conflict of interest

The authors declare that the research was conducted in the absence of any commercial or financial relationships that could be construed as a potential conflict of interest.

## Publisher’s note

All claims expressed in this article are solely those of the authors and do not necessarily represent those of their affiliated organizations, or those of the publisher, the editors and the reviewers. Any product that may be evaluated in this article, or claim that may be made by its manufacturer, is not guaranteed or endorsed by the publisher.

## Author disclaimer

The contents of this publication are solely the responsibility of the authors and do not represent the official views of these Institutions.
